# CT-based deep learning enables early postoperative recurrence prediction for intrahepatic cholangiocarcinoma

**DOI:** 10.1038/s41598-022-12604-8

**Published:** 2022-05-19

**Authors:** Taiichi Wakiya, Keinosuke Ishido, Norihisa Kimura, Hayato Nagase, Taishu Kanda, Sotaro Ichiyama, Kenji Soma, Masashi Matsuzaka, Yoshihiro Sasaki, Shunsuke Kubota, Hiroaki Fujita, Takeyuki Sawano, Yutaka Umehara, Yusuke Wakasa, Yoshikazu Toyoki, Kenichi Hakamada

**Affiliations:** 1 Department of Gastroenterological Surgery, Hirosaki University Graduate School of Medicine, 5 Zaifu-cho, Hirosaki City, Aomori 036-8562 Japan; 2 Department of Medical Informatics, Hirosaki University Hospital, Hirosaki City, Aomori 036-8562 Japan; 3 Hirosaki University School of Medicine, Hirosaki City, Aomori 036-8562 Japan; 4 Department of Surgery, Aomori Prefectural Central Hospital, Aomori City, Aomori 030-8553 Japan; 5 Department of Surgery, Aomori City Hospital, Aomori City, Aomori 0300821 Japan

**Keywords:** Computational biology and bioinformatics, Medical research

## Abstract

Preoperatively accurate evaluation of risk for early postoperative recurrence contributes to maximizing the therapeutic success for intrahepatic cholangiocarcinoma (iCCA) patients. This study aimed to investigate the potential of deep learning (DL) algorithms for predicting postoperative early recurrence through the use of preoperative images. We collected the dataset, including preoperative plain computed tomography (CT) images, from 41 patients undergoing curative surgery for iCCA at multiple institutions. We built a CT patch-based predictive model using a residual convolutional neural network and used fivefold cross-validation. The prediction accuracy of the model was analyzed. We defined early recurrence as recurrence within a year after surgical resection. Of the 41 patients, early recurrence was observed in 20 (48.8%). A total of 71,081 patches were extracted from the entire segmented tumor area of each patient. The average accuracy of the ResNet model for predicting early recurrence was 98.2% for the training dataset. In the validation dataset, the average sensitivity, specificity, and accuracy were 97.8%, 94.0%, and 96.5%, respectively. Furthermore, the area under the receiver operating characteristic curve was 0.994. Our CT-based DL model exhibited high predictive performance in projecting postoperative early recurrence, proposing a novel insight into iCCA management.

## Introduction

Primary liver cancer was the third leading cause of cancer death worldwide in 2020, with 830,180 deaths contributing to 8.3% of worldwide cancer-related deaths. In 2020, 905,677 liver cancer diagnoses were made globally, comprising 4.7% of worldwide cancer cases^1^. Intrahepatic cholangiocarcinoma (iCCA) is the second most common primary liver cancer after hepatocellular carcinoma and accounts for 10–15% of primary liver cancer^1^. Incidences of iCCA have been increasing over the last three decades^2,3^. However, the prognosis of iCCA, unfortunately, remains extremely poor, with a 5-year overall survival of 9%^4^. This cancer presents a substantial health problem worldwide, so treatments to improve survival are urgently needed.

In tackling this lethal disease, surgical resection has been the most fundamental and only treatment with the potential for cure^2,5^. Unfortunately, only about 20–40% of patients present with potentially operable disease^3,6^. In addition, recurrence following surgical resection of iCCA remains a big concern. Several studies have demonstrated that around 50% of patients recurred after curative-intent surgery^5,7,8^. In short, the number of patients who benefit from surgical resection alone is limited.

Preoperatively accurate evaluation of risk for postoperative recurrence contributes to maximizing the therapeutic success for iCCA patients. Several lines of evidence from the clinical studies have demonstrated that factors associated with a higher risk for recurrence include large tumor size, multiple tumors, vascular invasion, lymph node metastasis, and R1 resection^7–9^. These reports provided us with significant insight. However, definitive preoperative diagnosis of the above factors, especially lymph node metastasis, remains challenging^10^. Furthermore, these reports were analyzed using multivariable logistic regression modeling to identify the independent risk factors for postoperative recurrence. This method has been traditionally performed in clinical studies, but there have been certain limitations, such as selection of variables, confounding factors, and multicollinearity.

To resolve the above issues, in this study, we created a prediction model for early postoperative recurrence using artificial intelligence (AI). AI has the potential to revolutionize disease diagnosis and management in the medical field^11^. Deep learning (DL) has recently gained extensive attention as a technique for realizing the full potential of AI^12^. Convolutional neural networks (CNNs), which are a DL approach, are especially recognized as demonstrating high performance in image recognition^13,14^. Indeed, there have been some successful reports in applying DL to the assessment and prediction of radiological images in clinical settings^13,15,16^.

In the field of iCCA, a recent study using DL has demonstrated the feasibility of applying it to liver tumor diagnoses^17^. Moreover, Jeong et al. showed its usefulness in the prognostic estimation and stratification of susceptible individuals for adjuvant treatment after resection^18^. However, there has been no report applying DL to recurrence prediction in patients with iCCA after resection. Thus, this study aimed to investigate the potential of DL algorithms for predicting early postoperative recurrence through the use of preoperative iCCA images. Here, we have successfully developed a prediction model using CNNs and propose a novel concept in iCCA management from a completely different perspective.

## Methods

### Patients and study design

This multi-institutional, retrospective, observational study was approved by the Committee of Medical Ethics of Hirosaki University Graduate School of Medicine (Aomori, Japan; reference no. 2020-230-1). Informed consent was obtained in the form of opt-out on our website (https://www.med.hirosaki-u.ac.jp/hospital/outline/resarch/resarch.html), with the approval of the Committee of Medical Ethics of Hirosaki University Graduate School of Medicine. Our study did not include minors. This study was designed and carried out in accordance with the Declaration of Helsinki.

Between January 2001 and December 2019, 41 patients undergoing liver surgery for iCCA at three institutions were included in this study. Details of the surgical procedure are described in Supplemental content 1. All patients had a confirmed pathologic diagnosis. In this study, the exclusion criteria were as follows: patients who had not undergone CT examination before surgery or patients with missing postoperative course data. Recurrence after surgery was diagnosed using enhanced CT and/or MRI. In this study, early recurrence was defined as recurrence within a year after liver surgery.

### CT acquisition and tumor segmentation

**Figure 1:**
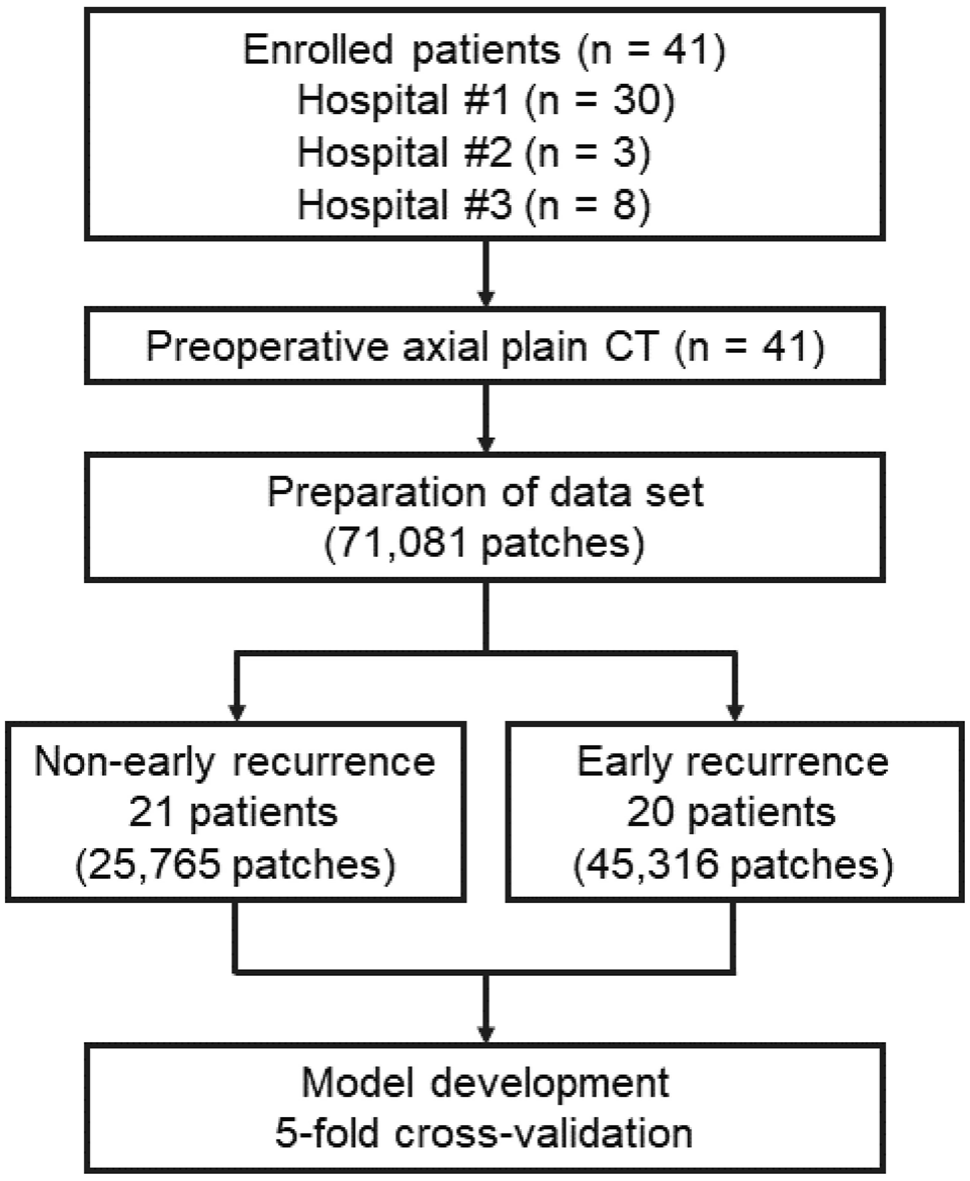
The study workflow and methodological process.

Our workflow is shown in Fig. 1. Preoperative axial plain CT images for each case were obtained from multiple institutions and were used for this study. Radiological assessment was performed by board-certified radiologists who were blind to the outcome of the patients. Board-certified surgeons and medical students performed CT acquisition and tumor segmentation based on the radiological assessment. Using a commercial viewer (ShadeQuest/ViewR, Fujifilm, Japan), the CT image showing the largest tumor area was selected. The entire tumor region was manually segmented with Adobe Illustrator and saved.

### Preparation of dataset

We trimmed a patch with a size of 128 × 128 pixels with 32 pixel stride from the entire segmented tumor area. Finally, 71,081 patches were obtained from 41 patients in the current study.

### Architecture of the CNN

ResNet50^19^ and Pytorch (a python library) were utilized (Available at: https://github.com/pytorch/pytorch). We did not use a pretrained model. The original acquired images of 128 × 128 pixels were converted into images of 224 × 224 pixels. We tuned the hyperparameters as follows: number of training epochs, 50; batch size, 128; learning rate, 0.00025 via trial and error; and number of outer layers, 2 classes. Cross-entropy was used as loss function and the Adam as optimizer.

### Evaluation methods

We used cross-validation to obtain more accurate results with less bias in the machine learning studies^20^. In this study, the dataset is split into five folds, one fold of which is for validation and the other folds are for training. The proportion of patients with versus without early recurrence was equal in each fold. The training and validation processes were repeated five times using different folds each time. The final results were then averaged and the standard deviation was calculated. The accuracy, sensitivity, specificity, positive predictive values, and negative predictive values were evaluated. The model was also evaluated using the area under the receiver operating characteristic (ROC) curve (AUC).

### Heatmap

The probability of early recurrence of the patches calculated by the trained model over the entire tumor area was modulated in gray scale from 0 to 255, which was assigned to pseudo-coloring; blue for a low-risk patch and red for a high-risk one on CT imaging. Heatmaps were generated by applyColorMap in OpenCV to visualize the most indicative region for the risk of early recurrence. Prediction of the probability of early recurrence was visualized by pseudo-coloring; COLORMAP_JET was applied to the grayscale CT images.

### Statistical analyses

Continuous variables were expressed as medians (ranges) and analyzed using nonparametric methods for non-normally distributed data (Mann–Whitney U-test). Categorical variables were reported as numbers (percentages) and analyzed using the chi-squared test or Fisher’s exact test, as appropriate. Variables with a significant relationship to early recurrence in univariate analysis were used in a binary logistic regression model. A difference was considered to be significant for values of *P* < 0.05. The statistical analyses were performed using IBM SPSS Statistics for Windows, Version 26.0 (IBM Corp, Armonk, NY, USA).

### Ethics approval and consent to participate

This study was approved by the Committee of Medical Ethics of Hirosaki University Graduate School of Medicine (Aomori, Japan; reference no. 2020–230-1). Informed consent was obtained in the form of opt-out on our website (https://www.med.hirosaki-u.ac.jp/hospital/outline/resarch/resarch.html), with the approval of the Committee of Medical Ethics of Hirosaki University Graduate School of Medicine. This study was designed and carried out in accordance with the Declaration of Helsinki.

### Consent for publication

Informed consent was obtained in the form of opt-out on our website (https://www.med.hirosaki-u.ac.jp/hospital/outline/resarch/resarch.html), with the approval of the Committee of Medical Ethics of Hirosaki University Graduate School of Medicine.

## Results

### Comparison of the perioperative characteristics of the non-early recurrence and early recurrence groups

The clinical characteristics of the 41 enrolled patients are shown in Table 1. Of the 41 patients, early recurrence was observed in 20 (48.8%). The early recurrence group demonstrated increased levels of tumor biomarkers such as carbohydrate antigen 19–9 and carcinoembryonic antigen (Table 1). However, there were no significant differences in the biomarkers between the groups. Univariate analysis indicated that Union for International Cancer Control (UICC) N category was the only significant predictor of early recurrence (*P* = 0.002). The odds ratio (OR) was 11.611 (95% confidence interval (CI): 2.116–63.726).

**Table 1:** Comparison of the perioperative characteristics of the non-early recurrence and early recurrence groups.

	All cases (n = 41)	Non-early recurrence (n = 21)	Early recurrence (n = 20)	*P* value
**Gender**				0.879
Male, n	20 (48.8)	10 (47.6)	10 (50.0)	
Female, n	21 (51.2)	11 (52.4)	10 (50.0)	
Age, year	69 (39–81)	72 (39–81)	68 (46–81)	0.388
Body height, cm	157.0 (133.0–178.0)	157.5 (133.0–177.0)	156.0 (140.5–178.0)	0.814
Body weight, kg	54.0 (34.0–81.6)	53.0 (34.0–81.6)	54.0 (38.9–73.5)	0.629
Body mass index, kg/m^2^	21.9 (16.6–29.3)	22.1 (17.0–29.3)	21.6 (16.6–27.4)	0.506
**Child–Pugh score**				0.232
A, n	39 (95.1)	21 (100)	18 (9.0)	
B, n	2 (4.9)	0	2 (10.0)	
C, n	0	0	0	
CA19-9, U/mL	152.0 (3.8–80,355.0)	128.6 (5.0–3120.0)	314.0 (3.8–80,355.0)	0.371
CEA, ng/mL	3.5 (0.5–61.6)	2.6 (0.5–43.1)	3.7 (0.5–61.6)	0.438
**Surgical procedure**				0.570
Right hepatectomy, n	12 (29.3)	5 (23.8)	7 (35.0)	
Left hepatectomy, n	18 (43.9)	9 (42.9)	9 (45.0)	
Right anterior sectionectomy, n	3 (7.3)	2 (9.5)	1 (5.0)	
Right posterior sectionectomy, n	1 (2.4)	0	1 (5.0)	
Partial resection, n	7 (17.1)	5 (23.8)	2 (10.0)	
Lymph node dissection, n	11 (26.8)	5 (23.8)	8 (40.0)	0.266
**Tumor factors**				
Tumor size, mm	50.0 (16.0–150.0)	50.0 (16.0–150.0)	55.0 (25.0–150.0)	0.347
Solitary tumor, n	28 (68.3)	17 (81.0)	11 (55.0)	0.074
**UICC 8th edition**				
T category, n				0.703
T1a	6 (14.6)	2 (9.5)	4 (20.0)	
T1b	3 (7.3)	2 (9.5)	1 (5.0)	
T2	24 (58.5)	14 (66.7)	10 (50.0)	
T3	5 (12.2)	2 (9.5)	3 (15.0)	
T4	3 (7.3)	1 (4.8)	2 (10.0)	
N category, n				0.002
N0	28 (68.3)	19 (90.5)	9 (45.0)	
N1	13 (31.7)	2 (9.5)	11 (55.0)	
M category, n				1.000
M0	41 (100)	21 (100)	20 (100)	
TNM Stage, n				0.055
IA	3 (7.3)	2 (9.5)	1 (5.0)	
IB	3 (7.3)	2 (9.5)	1 (5.0)	
II	17 (41.5)	12 (57.1)	5 (25.0)	
IIIA	3 (7.3)	2 (9.5)	1 (5.0)	
IIIB	15 (36.6)	3 (14.3)	12 (60.0)	
IV	0	0	0	
Intrahepatic metastasis, n	12 (29.3)	4 (19.0)	8 (40.0)	0.141
Positive surgical margin, n	3 (7.3)	2 (9.5)	1 (5.0)	> 0.999

*CA19-9* carbohydrate antigen 19-9, *CEA* carcinoembryonic antigen, *UICC* union for international cancer control.

### Binary logistic regression analysis

To predict early recurrence, we performed a binary logistic regression analysis, which is one of the traditional methods. We set early recurrence as the dependent variable. N category, which was found as a significant predictor through univariate analysis, was entered into a binary logistic regression analysis. Furthermore, we entered the UICC T category and surgical margin status, which have been reported as factors associated with a higher risk for recurrence.

**Figure 2:**
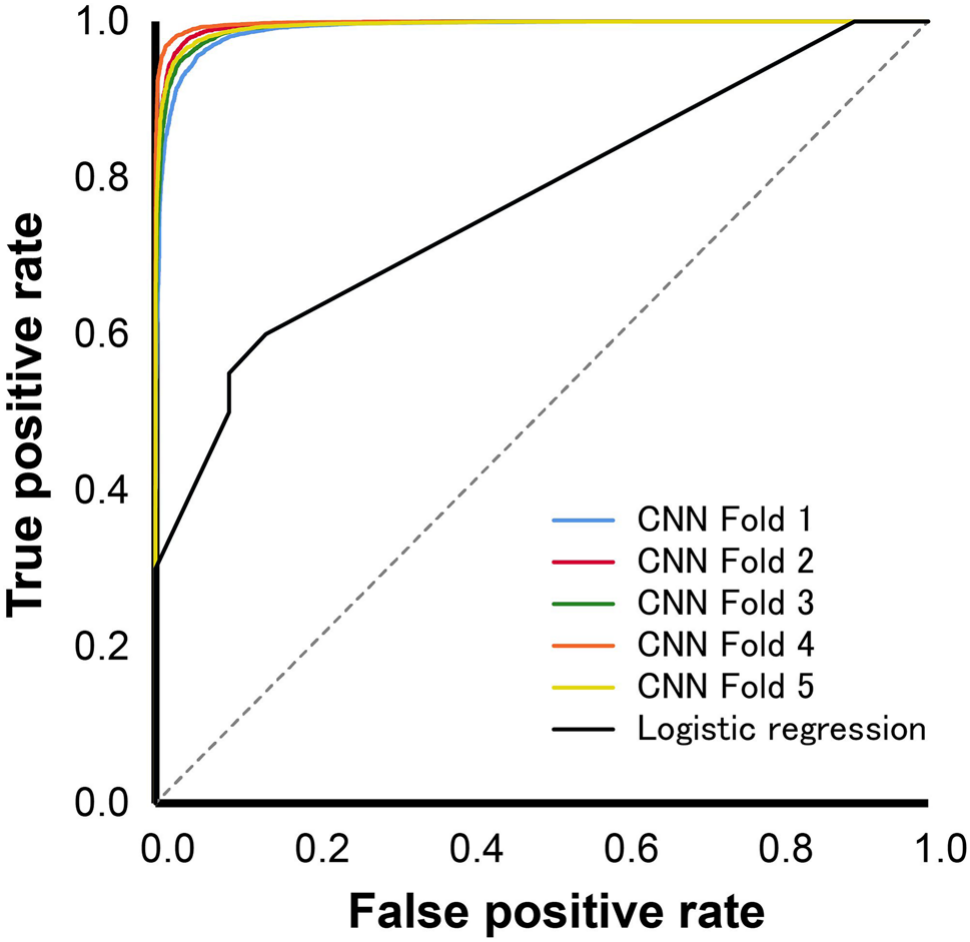
The receiver operating characteristics curves of logistic regression analysis and the DL model. ROC curves show the performance of logistic regression analysis and the ResNet model in the validation dataset in detecting early recurrence. The AUC of logistic regression analysis is 0.770, and the average AUC of the convolutional neural network (CNN) model is 0.994.

Binary logistic regression indicated that UICC N category was a significant predictor of early recurrence (Chi-Square = 11.952, and *P* = 0.063). The result of the Hosmer–Lemeshow test was *P* = 0.879. UICC N category was significant at the 5% level (Wald = 7.430, *P* = 0.006). The OR was 11.840 (95% CI: 2.002–70.008). The model correctly predicted 85.7% of cases without early recurrence and 60.0% of cases with early recurrence, giving an overall correct prediction rate of 73.2%. The model achieved an AUC of 0.770 (95% CI: 0.624–0.916) (Fig. 2).

### Performance of the ResNet50 model

A total of 25,765 patches were obtained from the 21 patients without early recurrence. Furthermore, a total of 45,316 patches were obtained from the 20 patients with early recurrence after surgery. Finally, a total of 71,081 patches were extracted from the 41 patients in the current study.

The average accuracy of the ResNet model for predicting early recurrence was 98.2% for the training dataset. The average sensitivity, specificity, and positive and negative predictive values were 98.9%, 97.0%, 98.3%, and 98.0%, respectively (Table 2). The model achieved an AUC of 0.9983 (95% CI: 0.9982–0.9985) in the training dataset.

**Table 2:** Performance of the DL model in the training data set.

	Fold 1	Fold 2	Fold 3	Fold 4	Fold 5	Average	SD
Sensitivity, %	99.0	98.5	99.0	99.6	98.4	98.9	0.5
Specificity, %	94.4	97.9	96.6	99.1	97.1	97.0	1.7
False negative rate, %	1.0	1.5	1.0	0.4	1.6	1.1	0.5
False positive rate, %	5.6	2.1	3.4	0.9	2.9	3.0	1.7
Positive predictive value, %	96.9	98.8	98.1	99.5	98.4	98.3	1.0
Negative predictive value, %	98.1	97.5	98.2	99.3	97.1	98.0	0.9
Accuracy, %	97.3	98.3	98.1	99.4	97.9	98.2	0.8
AUC	0.997	0.999	0.998	1.000	0.998	0.998	0.1

*AUC* the area under the receiver operating characteristic curve, *SD* standard deviation.

Likewise, the model showed high predicting performance in the validation dataset. The average sensitivity, specificity, and positive and negative predictive values were 97.8%, 94.0%, 96.7%, and 96.1%, respectively. In the validation dataset, the ResNet model achieved an accuracy of 96.5% (Table 3). The model achieved an AUC of 0.994 (95% CI: 0.993–0.995) in the validation dataset (Fig. 2).

**Table 3:** Performance of the DL model in the validation data set.

	Fold 1	Fold 2	Fold 3	Fold 4	Fold 5	Average	SD
Sensitivity, %	97.9	97.6	97.8	98.4	97.5	97.8	0.3
Specificity, %	90.6	95.9	92.5	96.6	94.6	94.0	2.5
False negative rate, %	2.1	2.4	2.2	1.6	2.5	2.2	0.3
False positive rate, %	9.4	4.1	7.5	3.4	5.4	6.0	2.5
Positive predictive value, %	94.8	97.7	95.8	98.1	96.9	96.7	1.3
Negative predictive value, %	96.2	95.8	95.9	97.1	95.5	96.1	0.6
Accuracy, %	95.3	97.0	95.9	97.7	96.4	96.5	1.0
AUC	0.990	0.996	0.993	0.998	0.994	0.994	0.3

*AUC* the area under the receiver operating characteristic curve, *SD* standard deviation.

### Highlighting areas with the risk of early recurrence by heatmap

**Figure 3:**
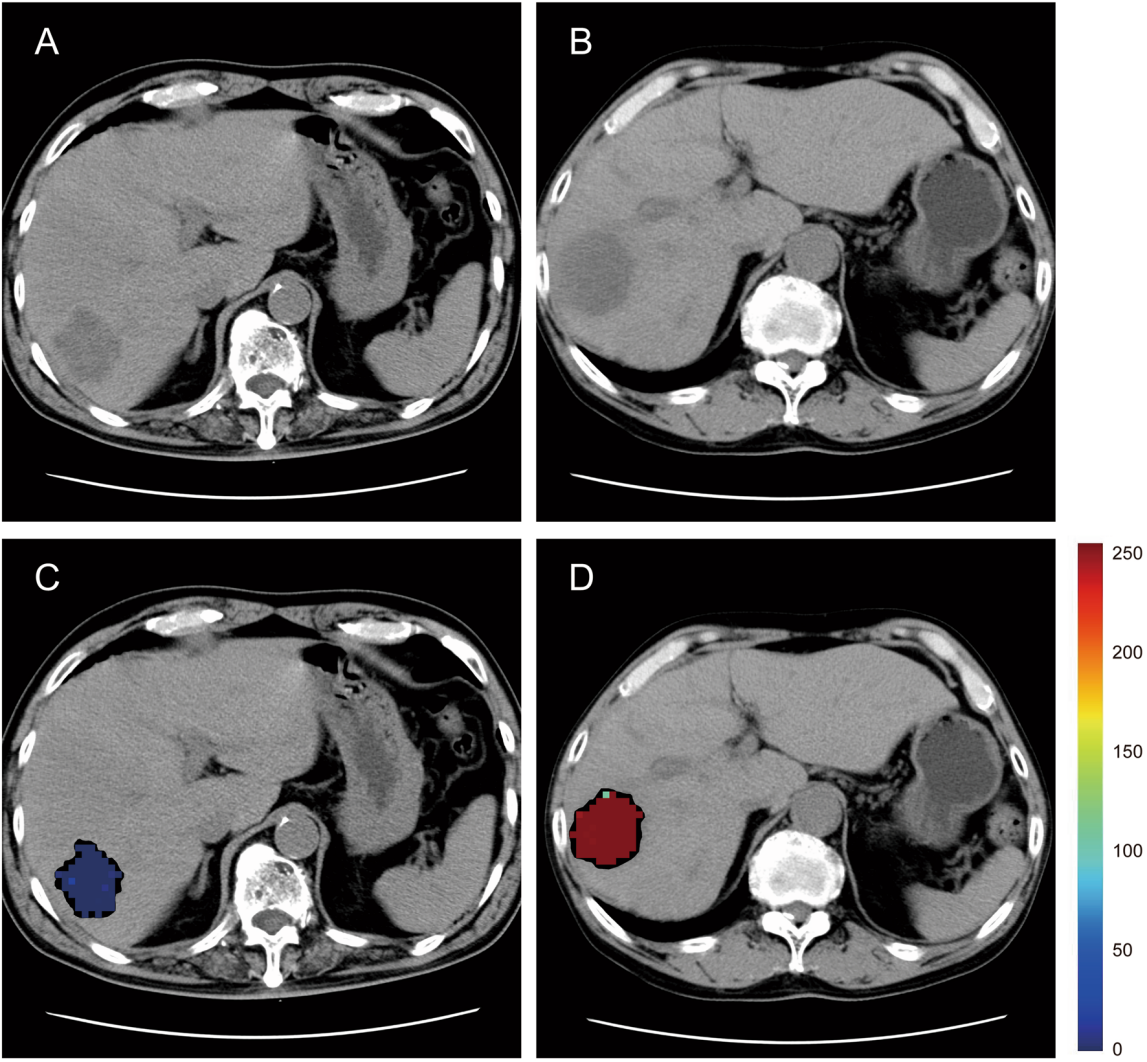
A heatmap of iCCA on a preoperative plain CT using our prediction model. The color bar illustrates the degree of probability the model paid to it. Red areas represent a high risk of early recurrence; blue areas represent a low risk of early recurrence. (**A**) original image of a non-early recurrence case; (**B**) original image of an early recurrence case; (**C**) heatmap of a non-early recurrence case; (**D**) heatmap of an early recurrence case.

A representative heatmap of the tumor area on a preoperative plain CT based on probability calculated using our prediction model is shown in Fig. 3. The heatmap can be superimposed on the input image to highlight the areas the model considers important in making its diagnosis. In short, the heatmap can contribute to assisting physicians before surgery by highlighting areas with the risk of early recurrence.

### Factors that can influence misprediction

We further investigated factors that can influence misprediction. Using our own model, we calculated the prediction accuracy of each case based on the patches in each one. In this study, we defined cases with prediction accuracy of the first quartile or less (96.0%), as occurrences of misprediction. Table 4 reveals a comparison of the perioperative characteristics of the cases with an accuracy of 96.0% or less and the cases with over 96.0% accuracy. Univariate analysis showed that smaller tumor size was a significant factor in misprediction (*P* = 0.025). Likewise, looking at tumor size only, also showed a significant difference in the comparison of the two groups when divided by the median value of prediction accuracy (97.8%). Collectively, these data suggested that our prediction model can perform excellently, particularly with larger iCCA.

**Table 4:** Comparison of perioperative characteristics depending on prediction accuracy.

	Under 25% (n = 11)	Over 25% (n = 30)	*P* value
**Gender**			0.159
Male, n	3 (27.3)	17 (56.7)	
Female, n	8 (72.7)	13 (43.3)	
Age, year	68 (60–77)	70 (39–81)	0.755
Body height, cm	154.0 (133.0–173.5)	158.5 (140.5–178.0)	0.333
Body weight, kg	51.0 (34.0–73.5)	55.3 (38.0–81.6)	0.547
Body mass index, kg/m^2^	22.1 (19.2–26.0)	21.7 (16.6–29.3)	0.937
**Child–Pugh score**			0.380
A, n	11 (100)	28 (93.3)	
B, n	0	2 (6.7)	
C, n	0	0	
CA19-9, U/mL	39.1 (5.0–3120.0)	179.0 (3.8–80,355.0)	0.526
CEA, ng/mL	4.1 (0.5–43.1)	3.0 (0.5–61.6)	0.706
**Surgical procedure**			0.661
Right hepatectomy, n	3 (27.3)	9 (30.0)	
Left hepatectomy, n	5 (45.5)	13 (43.3)	
Right anterior sectionectomy, n	0	3 (10.0)	
Right posterior sectionectomy, n	0	1 (3.3)	
Partial resection, n	3 (27.3)	4 (13.3)	
Lymph node dissection, n	4 (36.4)	9 (30.0)	0.698
**Tumor factors**			
Tumor size, mm	42.0 (16.0–60.0)	57.5 (17.0–150.0)	0.025
Solitary tumor, n	8 (72.7)	20 (66.7)	> 0.999
**UICC 8th edition**			
T category, n			0.482
T1a	1 (9.1)	5 (16.7)	
T1b	0	3 (10.0)	
T2	8 (72.7)	16 (53.3)	
T3	2 (18.2)	3 (10.0)	
T4	0	3 (10.0)	
N category, n			> 0.999
N0	8 (72.7)	20 (66.7)	
N1	3 (27.3)	10 (33.3)	
M category, n			1.000
M0	11 (100)	30 (100)	
TNM Stage, n			0.406
IA	1 (9.1)	2 (6.7)	
IB	0	3 (10.0)	
II	5 (45.5)	12 (40.0)	
IIIA	2 (18.2)	1 (3.3)	
IIIB	3 (27.3)	12 (40.0)	
IV	0	0	
Intrahepatic metastasis, n	3 (27.3)	9 (30.0)	> 0.999
Positive surgical margin, n	1 (9.1)	2 (6.7)	> 0.999
Early recurrence, n	4 (36.4)	16 (53.3)	0.484
Prediction accuracy, %	95.4 (85.6–96.0)	98.4 (96.2–100)	< 0.001

*CA19-9* carbohydrate antigen 19-9, *CEA* carcinoembryonic antigen, *UICC* union for international cancer control.

## Discussion

We applied the DL model to predict postoperative early recurrence of iCCA. We have successfully demonstrated high performance in the prediction of early postoperative recurrence using plain preoperative CT images. The accuracy of the DL model far exceeded that of the binary logistic regression analysis (AUC, 0.994 vs. 0.770). This report represents the first study in which a DL model based on CT images is used to predict early postoperative recurrence in iCCA. Our results may yield a novel insight into personalized treatment strategies, including neoadjuvant and adjuvant chemotherapy, in iCCA management.

Adjuvant chemotherapy is certainly expected to increase the survivorship of patients with iCCA^21–23^. Isolating results has been a challenge as past prospective randomized trials have included not only iCCA but also other bile duct cancers^23–25^. Furthermore, the indication for adjuvant chemotherapy in those studies was heterogeneity. In short, the selection criteria of susceptible individuals for adjuvant chemotherapy is not well established. To address this issue, Jeong et al. showed the usefulness of an AI framework in the prognostic estimation and stratification of susceptible individuals for adjuvant treatment after resection in iCCA patients. Though they reported applying DL to recurrence prediction, they did not use CT and did not specifically predict early recurrence. In contrast, we intended to predict early postoperative recurrence directly by CT. Our model, which can directly predict early recurrence, would be used to predict who should receive adjuvant chemotherapy based on their risk of recurrence.

Liang et al. conducted a single-center retrospective study and built a radiomics nomogram to predict early recurrence of iCCA after surgical resection^26^. Their nomogram, using preoperative arterial-phase contrast-enhanced magnetic resonance imaging (MRI), achieved an AUC of 0.82 and 0.77 in the training and validation cohorts, respectively. They used manual engineered features and selected the earlies recurrence-related features using a least absolute shrinkage and selection operator logistic regression analysis. Zhao et al. used radiomics from MRI to predict early recurrence. Their radiomics model showed a preferable predictive performance (AUC 0.889)^27^. Compared with the previous radiomics model using MRI, our model, which is based on DL features, achieved higher predictive performance (AUC 0.994).

Based on our results, which perform in such a highly predictive manner with the model addressing postoperative early recurrence, we propose a new concept in iCCA management. Though we need to discuss further which population, patients, those with or without early recurrence, is fit for adjuvant chemotherapy, achieving quite high levels of predictive accuracy, compared to conventional methods, can provide valuable information for determining adjuvant therapy and developing surgical plans, thereby facilitating pretreatment decisions. Moreover, this model can help optimize postoperative surveillance intervals for early detection of recurrence based on the risk of early recurrence.

Thanks to the advantages reaped from DL, we physicians, can easily apply computer-aided diagnosis^16,28^. Deep learning algorithms, such as CNN, have been widely used in the field of image diagnosis and prediction owing to their being fast, accurate, and reproducible^28,29^. CNN can uncover details in medical images that human experts cannot find, and automatically render a quantitative assessment^30^. Generally, even expert radiologists and surgeons cannot always access meaningful findings that would enable physicians to decide on a treatment strategy from plain CT images. In fact, there have been no reports or guidelines that recommend using plain CT images for risk assessment of postoperative recurrence in iCCA. Several lines of evidence, including our study, can lead to a paradigm shift in the recognition of AI in the field of iCCA treatment.

The present study has several limitations. This is a retrospective study. In addition, although this is a pilot study, the patient population was small. Part of the reason is because it is still challenging to detect iCCA at an early stage. Patients are often diagnosed in advanced stages, not indicated for surgery. As a result, the number of iCCA patients included in this study, which focused on recurrence after surgery, was low. However, our model achieved high predictive performance. If we had access to additional training data from a large cohort, we could achieve even higher prediction accuracy and generality. To establish clinical applications, sufficient datasets are fundamental requirements. A novel AI approach based on analyzing a huge database, such as national or regional datasets, would be attractive to both clinicians treating iCCA and their patients. An accurate and robust prediction model can ultimately contribute to a better prognosis in iCCA patients. We expect that future studies will expand this approach.

There was the question of possible lack of homogeneity in CT techniques over the past 20 years that has been a point of contention (Supplementary Table 1). Nevertheless, our model achieved high predictive performance. These results suggested that relative heterogeneity of CT techniques may not be a big issue because of the handling of huge information from CT images through DL. Certainly, homogeneity of CT techniques would be preferable. However, it would not be practical in a real clinical setting for all patients to undergo CT exams using the same scanner and technique. In short, the use of a diverse set of CT acquisitions was not a limitation, it was a benefit to the study.

In conclusion, our DL model, using plain preoperative CT images of iCCA, exhibited high predictive performance in projecting postoperative early recurrence. The present multicenter study has provided a novel approach to predict early recurrence after surgery. This model may help clinicians in the selection of patients for neoadjuvant and/or adjuvant therapy. Furthermore, this model may help optimize risk-based postoperative surveillance intervals for early detection of recurrence. In short, this approach can contribute to personalized strategies in iCCA treatment. To establish a clinical application, conducting a study using a huge dataset, such as national dataset, is the hope for the future.

## Supplementary Information

Supplementary Information 1.

Supplementary Information 2.

## Data Availability

The data generated or analyzed during this study are included in this published article and its supplementary information files. Some datasets generated and/or analyzed during the current study are not publicly available due to privacy but are available from the corresponding author on reasonable request.
