# Supplementary material for: CT-based deep learning enables early postoperative recurrence prediction for intrahepatic cholangiocarcinoma
Source: Sci Rep. 2022 May 19;12:8428. doi: 10.1038/s41598-022-12604-8 (PMC9120508; doi:10.1038/s41598-022-12604-8)
Supplement: Supplementary file 1 — Supplementary Information 1. [file 41598_2022_12604_MOESM1_ESM.docx]

**Supplemental Content 1**

***Surgical procedures***

The tumor location was judged from preoperative imaging such as computed tomography or magnetic resonance imaging. The tumors were classified as hilar or peripheral type. We selected the type of liver resection based on the tumor location. Lymph node dissection was usually performed for the hilar type cases. For the peripheral type, lymph node sampling was performed in patients with known or suspected nodal involvement. When it was determined that sufficient surgical margins could be obtained, partial resection was selected. Cholecystectomy was performed selectively only when necessary to secure the surgical field.
