# Supplementary material for: CT-based deep learning enables early postoperative recurrence prediction for intrahepatic cholangiocarcinoma
Source: Sci Rep. 2022 May 19;12:8428. doi: 10.1038/s41598-022-12604-8 (PMC9120508; doi:10.1038/s41598-022-12604-8)
Supplement: Supplementary file 2 — Supplementary Information 2. [file 41598_2022_12604_MOESM2_ESM.docx]

**Supplementary table 1.** Information of CT scanners.

| Case | Brand | Scanner |
| --- | --- | --- |
| Hospital A-1 | GE Healthcare | Discovery CT750 HD |
| Hospital A-2 | TOSHIBA | Aqulion ONE |
| Hospital A-3 | GE Healthcare | Discovery CT750 HD |
| Hospital A-4 | SIEMENS | SOMATOM Definition |
| Hospital A-5 | TOSHIBA | Aqulion ONE |
| Hospital A-6 | TOSHIBA | Aqulion ONE |
| Hospital A-7 | TOSHIBA | Aqulion ONE |
| Hospital A-8 | SIEMENS | SOMATOM Definition |
| Hospital A-9 | GE Healthcare | Discovery CT750 HD |
| Hospital A-10 | SIEMENS | SOMATOM Definition |
| Hospital A-11 | GE Healthcare | Discovery CT750 HD |
| Hospital A-12 | TOSHIBA | Aqulion ONE |
| Hospital A-13 | TOSHIBA | Aqulion ONE |
| Hospital A-14 | TOSHIBA | Aqulion ONE |
| Hospital A-15 | GE Healthcare | Discovery CT750 HD |
| Hospital A-16 | GE Healthcare | Discovery CT750 HD |
| Hospital A-17 | TOSHIBA | Aqulion ONE |
| Hospital A-18 | SIEMENS | Definition |
| Hospital A-19 | SIEMENS | Definition |
| Hospital A-20 | GE Healthcare | Lightspeed Qx/i |
| Hospital A-21 | GE Healthcare | Lightspeed Qx/i |
| Hospital A-22 | GE Healthcare | Lightspeed Qx/i |
| Hospital A-23 | GE Healthcare | Lightspeed Qx/i |
| Hospital A-24 | GE Healthcare | Lightspeed Qx/i |
| Hospital A-25 | GE Healthcare | Lightspeed Qx/i |
| Hospital A-26 | GE Healthcare | Lightspeed Qx/i |
| Hospital A-27 | GE Healthcare | Lightspeed Qx/i |
| Hospital A-28 | GE Healthcare | Lightspeed Qx/i |
| Hospital A-29 | GE Healthcare | Lightspeed Qx/i |
| Hospital A-30 | GE Healthcare | Lightspeed Qx/i |
| Hospital B-1 | TOSHIBA | Aqulion |
| Hospital B-2 | TOSHIBA | Aqulion |
| Hospital B-3 | TOSHIBA | Aqulion |
| Hospital C-1 | GE Healthcare | Lightspeed |
| Hospital C-2 | GE Healthcare | Lightspeed |
| Hospital C-3 | GE Healthcare | Lightspeed |
| Hospital C-4 | GE Healthcare | Lightspeed |
| Hospital C-5 | GE Healthcare | Discovery CT750 HD |
| Hospital C-6 | GE Healthcare | Discovery CT750 HD |
| Hospital C-7 | GE Healthcare | Discovery CT750 HD |
| Hospital C-8 | GE Healthcare | Optima CT660 |

CT, computed tomography
